# Oral Health Scales: Design of an Oral Health Scale of Infectious Potential

**DOI:** 10.4317/medoral.18427

**Published:** 2013-03-25

**Authors:** Marta Relvas, Pedro Diz, Juan Seoane, Inmaculada Tomás

**Affiliations:** 1 Associate Lecturer. Department of Dental Sciences. Higher Institute of the Health Sciences-North, Oporto, Portugal; 2 Lecturers. Stomatology Department. Faculty of Medicine and Dentistry. Santiago de Compostela University, Spain

## Abstract

Objectives: In this paper we propose a new Global Oral Health Scale that will allow the infectious potential of the oral cavity, clinically manifest as local and focal infections, to be condensed into a single parameter. Study Design: Based on a number of oral health scales previously designed by our group, we designed a final version that incorporates dental and periodontal variables (some of them evaluated using corroborated objective indices) that reflect the presence of caries and periodontal disease. Results: The application of the proposed oral health scale requires the examination of 6 sites per tooth (mesio-buccal, medio-buccal, disto-buccal, disto-lingual, medio-lingual and mesio-lingual). The following variables are analysed: number of tooth surfaces with supragingival plaque, determined using the O’Leary index; number of teeth with caries and the severity of the caries; number of tooth surfaces with gingival inflammation, determined using the Ainamo and Bay index; and number of tooth surfaces with pockets ?4 mm and severity of the pockets. These variables are then grouped into 2 categories, dental and periodontal. The final grades of dental and periodontal health correspond to the grades assigned to a least 2 of the 3 variables analysed in each of these categories. The category (dental or periodontal) with the highest grade is the one that determines the grade of the Global Oral Health Scale. Conclusion: This scale could be particularly useful for the epidemiological studies comparing different populations and for analysis of the influence of distinct degrees of oral health on the development of certain systemic diseases.

** Key words:**Scale, oral health, infectious potential, systemic disease.

## Introduction

Periodontal disease and caries, the principal causes of tooth extractions in adults (1,2), are the most common chronic diseases in the general population. They have marked repercussions in terms of public health, because of their prevalence, their impact on the individual and on the population as a whole, and the inherent cost of treatment which, in some countries, is the fourth largest component of the health budget. Oral health has physical, psychological and social consequences as it is directly implicated in phonation, mastication and facial appearance and also indirectly affects growth and social well-being (3,4).

Caries and periodontal disease are infectious diseases associated with bacterial colonisation of the tooth surfaces (the biofilm). Factors such as bacterial specificity and pathogenicity and individual susceptibility can influence the onset, pattern of progression and clinical characteristics of oral diseases associated with the biofilm (5).

In recent years, numerous authors have suggested that there is an association between oral infections (particularly in the form of periodontal disease) and an increased risk of developing certain systemic diseases (6,7). The most notable related diseases are those affecting the cardiovascular (8) and respiratory (9) systems, though further diseases have recently been added to this list, such as diabetes, rheumatoid arthritis, osteoporosis, cancer of the pancreas, metabolic syndrome, renal failure and even degenerative conditions such as Alzheimer’s disease (10). There is also an increased risk of premature birth (11).” 

In the dental literature there are few publications describing the combination of different variables to create health scales. The idea of representing the state of oral health in a single numerical value is of particular interest with the development of the so-called “periodontal medicine”, which consists of establishing correlations between infectious or inflammatory disorders of the oral cavity and the onset of certain systemic diseases (6,7). For this reason, some authors have designed specific oral health scales, such as the Total Dental Index (12), the modified Total Dental Index (13,14), the Dental Asymptotic Score (15) and the Brief Oral Health Status Examination (BOHSE) (16) ( Table 1, Table 2).

**Table 1:**
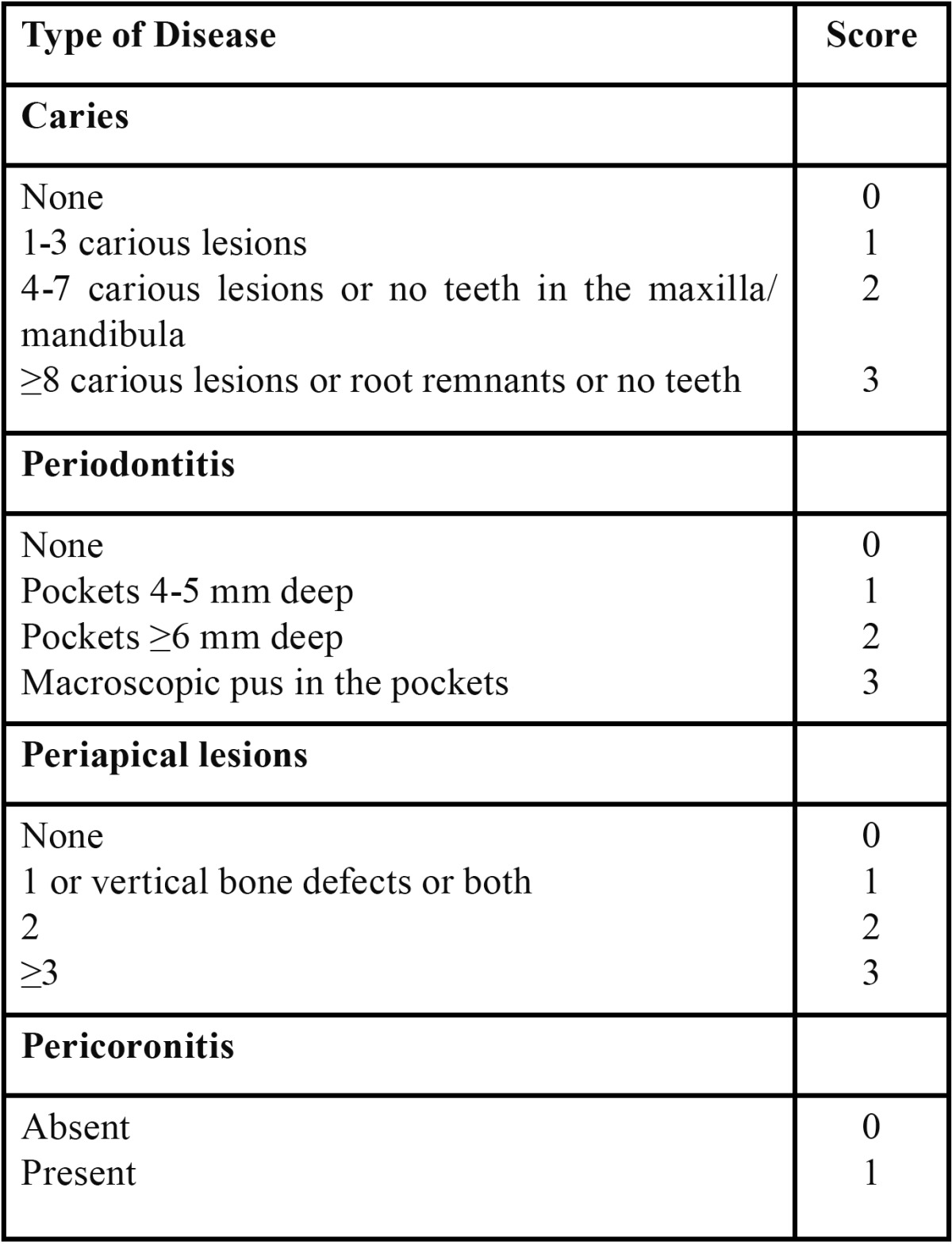
SORT Criteria (Strength of Recommendation Taxonomy).

**Table 2:**
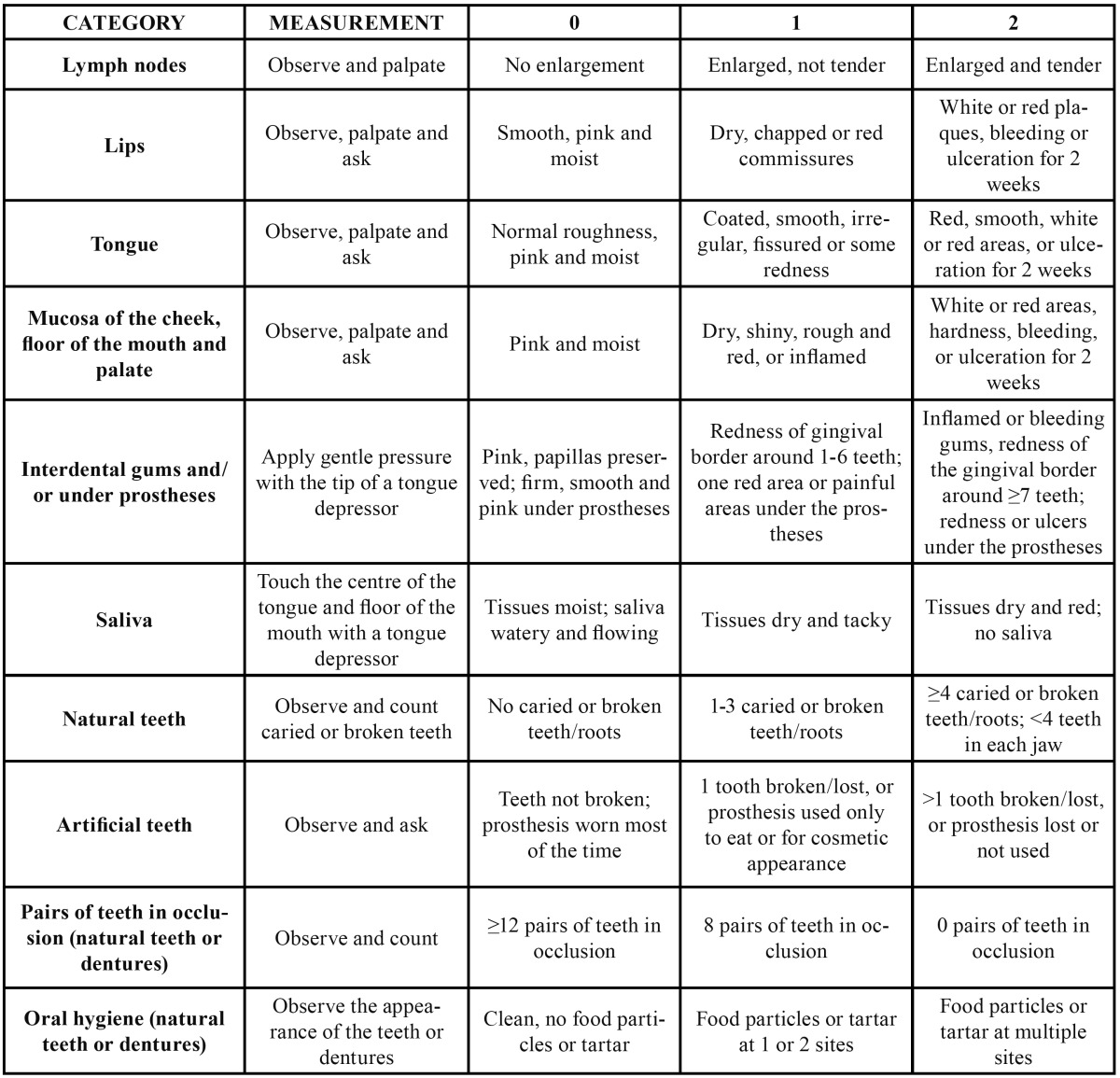
Brief Oral Health Status Examination ( BOHSE) (16).

In this paper we propose a new Global Oral Health Scale that enables the infectious potential of the oral cavity, manifest clinically as local and focal infections, to be condensed into a single parameter.

## Material and Methods

The first oral health scale designed by our group was created in 1997 in the context of a prospective study to evaluate the oral health status of a group of oncology patients undergoing radiotherapy treatment in the maxillofacial area (unpublished data). Patients underwent dental evaluation based on the DMFT index (sum of permanent teeth with caries, missing teeth and permanent filled teeth), performed using a conventional probe and mirror. The supragingival plaque and calculus were assessed by direct observation using the following categories: 0, absence of plaque/calculus; 1, plaque/calculus on the gingival third; 2, plaque/calculus on the gingival and medial thirds; 3, plaque/calculus on the whole vestibular surface. Subgingival plaque and calculus were detected using a conventional periodontal probe, registering its presence (grade 1) or absence (grade 0). Tooth mobility was determined using a probe and a mirror handle with which the tooth was tapped in a vestibular-lingual direction. Mobil-ity was classified into 4 grades: 0, normal, no mobility; 1, minimal mobility; 2, visible mobility (?1 mm); 3, marked instability (>1 mm). The depth of the periodontal pockets was determined using a World Health Organization (WHO) CP12 periodontal probe. Pockets were classified into 3 groups: ?3 mm; 4-5 mm; ?6 mm. Gingival bleeding was evaluated by sliding a blunt periodontal probe from the base of the papilla to its most prominent point, with the following scores: 0, no bleeding; 1, bleeding on probing; 2, spontaneous bleeding.

An oral health scale with a range from grade 0 (“healthy mouth”) to 3 (“very unhealthy mouth”) was drawn up to analyse the overall significance of the intraoral findings. The DMFT values, the presence of supragingival and subgingival plaque, dental mobility, depth of the periodontal pockets and the presence or absence of gingival bleeding were included in that analysis. The grade assigned to each patient corresponded to the one that coincided with the majority of the values obtained.

A few years later, aware of the limitations of the initial scale, we developed a new Global Oral Health Scale, which we applied to a collective of patients awaiting tooth extraction. Our aim was to determine whether the oral health status could affect the prevalence, duration or aetiology of bacteraemia provoked by the dental manipulation (17). This new scale included dental and periodontal health criteria ( Table 3). The following dental variables were analysed: accumulation of supragingival plaque, assessed using the simplified Green and Vermilion index (18); absolute number of caries; and presence of submucous abscesses and/or periapical foci. The periodontal variables included the following: calculus accumulation, assessed using the Ramfjord calculus index (19); gingival information, evaluated using the Löe and Silness index (20); depth of periodontal pockets; and tooth mobility, assessed using the Ramfjord dental mobility index (19).

**Table 3:**
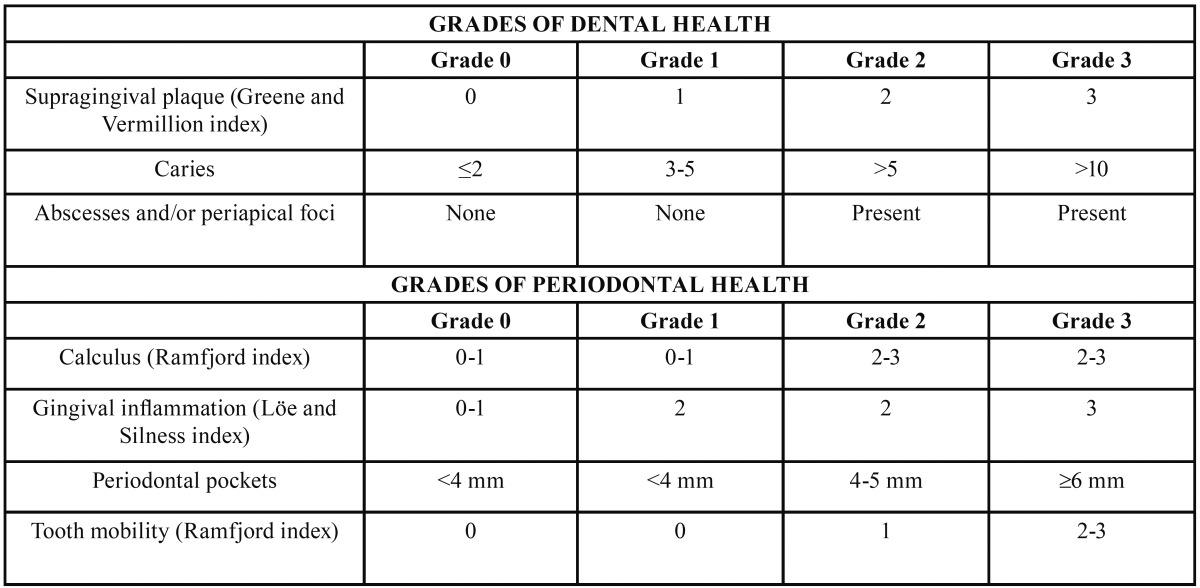
Modified Version of the Authors’ Global Oral Health Scale (17).

The designated grade of dental health corresponded to the grade assigned to at least 2 of the 3 dental variables analysed. In cases in which different grades were assigned to each of the 3 variables analysed, the grade used is the one that corresponds to “caries”. The designated grade of periodontal health corresponded to the grade assigned to at least 3 of the 4 periodontal variables analysed, except in children, in whom tooth mobility was not evaluated. In those cases in which different grades were assigned to each of the 4 variables analysed, the grade used is the one that corresponds to “periodontal pockets”. The grade of the Global Oral Health Scale corresponded to the higher of the grades finally assigned to dental and periodontal health ( Table 3).

The incorporation of corroborated indices increased the reproducibility of the revised version of the Global Oral Health Scale and reduced inter-examiner and intra-examiner variability. We were thus able to use the scale in subsequent research on bacteraemia of oral origin (21). That revised version of the Global Oral Health Scale also had certain limitations and we have progressively included modifications, which have led to the current proposal. Application of the Global Oral Health Scale proposed in this paper requires compliance with certain inclusion criteria: age 15 years or older and the presence of 24 or more teeth in the oral cavity.

## Results

The proposed oral health scale requires the examination of 6 sites per tooth: mesio-buccal, medio-buccal, disto-buccal, disto-lingual, medio-lingual and mesio-lingual. Maintaining the structure of the previous scale, the recorded variables are grouped into 2 categories, dental and periodontal. The following variables are analysed: number of tooth surfaces with supragingival plaque, assessed using the O’Leary index (22); number of teeth with caries, diagnosed using a probe and mirror, and severity of the caries (1, enamel; 2, enamel and dentine; 3, enamel, dentine and pulp); number of tooth surfaces with gingival inflammation, assessed using the Ainamo and Bay index (23); number of tooth surfaces with pockets ?4 mm, and the severity of the pockets (mean depth of the periodontal pockets, determined using a manual periodontal probe calibrated at 3, 6, 8 and 11 mm (PCP 11, Hu-Frieday, Chicago, Illinois, USA).

The designated grades of dental health and periodontal health correspond to the grade assigned to at least 2 of the 3 variables analysed in each of the respective categories. When different grades are assigned to each of the 3 variables in each category, the grade used is the one that corresponds to “caries” for dental health and to “pathological pockets ?4 mm” for periodontal health. If the same grade is assigned to 2 variables in one category and the grade of the third variable is 2 levels higher, the grade assigned to the category is the grade immediately above the grade of the coinciding variables. The category (dental or periodontal) with the highest grade is the one that determines the grade of the Global Oral Health Scale ( Table 4).

**Table 4:**
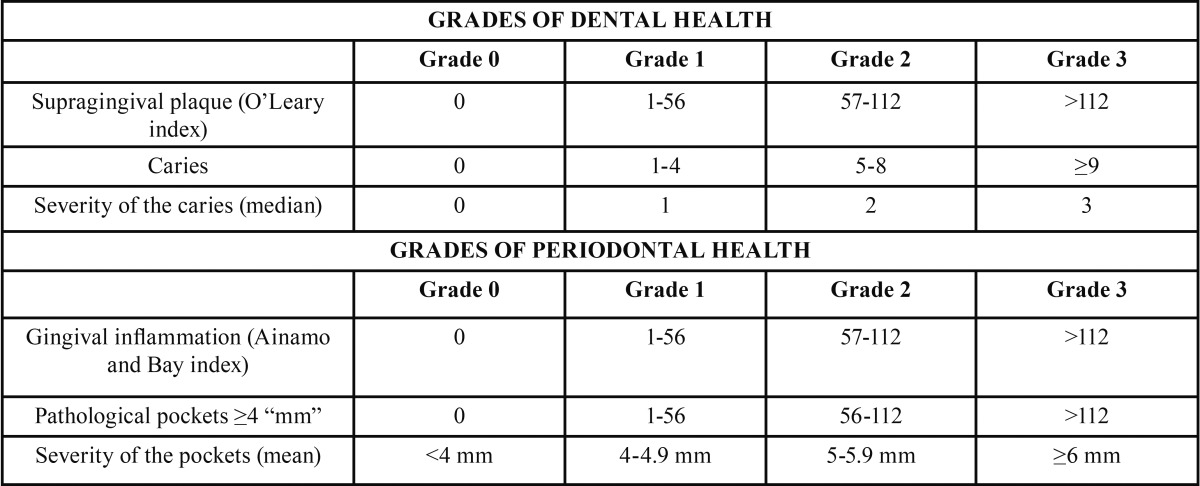
Updated Version of the Authors’ Global Oral Health Scale.

Although all the teeth present in the mouth were examined for the initial design of the scale, the reliability of half-mouth partial examination systems that combine one superior and inferior quadrant reduces the complexity of the evaluation process and, therefore, the time of application of the scale and its cost (24).

## Discussion

Mattila et al. (12) developed the Total Dental Index, the first oral health scale, to compare the oral health of a group of patients after myocardial infarction with that of a control group. That index analysed the presence of caries, periodontal disease, periapical lesions and pericoronitis ( Table 1). The Total Dental Index score was obtained from the sum of the values recorded for each variable, giving a final score between 0 and 10, with a higher score indicating more severe disease. Those authors also described the “Orthopantomographic Index”, which was based on an assessment of periapical lesions, lesions caused by tertiary caries, vertical bone loss, the presence of radiolucent areas at the furcation and lesions caused by pericoronitis (12). The Total Dental Index had certain limitations, such as the inclusion of edentulous jaws, which could affect oral health in functional terms but which has a lower infectious potential than remaining natural teeth. In addition, some of the variables recorded required a panoramic x-ray to be performed, which limited its use in epidemiological studies.

In 1997, Grau et al. (25) performed a case-control study to investigate whether recurrent chronic infections of the respiratory tract, of the ear, nose and throat or of the oral cavity constituted risk factors for cerebrovascular ischaemia. Those authors used a modified Total Dental Index to evaluate all patients (13,14). The innovations in that index consisted of the introduction of a new variable called “non-vital teeth without periapical lesions” (0, none; 1, present with endodontia; 2, present without endodontia) and an increase in the score for pericoronitis to 3 points instead of 1, making the maximum attainable score 14 points instead of 10 in the original index.

Recently, Oikarinen et al. (26) used the Total Dental Index to investigate a potential relationship between coronary artery disease and infectious odontogenic conditions detectable on x-ray. The authors used the original index with minor modifications to minimise its drawbacks: exclusion of edentulous patients, of the macroscopic detection of pus within periodontal pockets and of vertical bone loss.

Janket et al. (15) postulated that the combination of several oral lesions that stimulated the production of inflammatory mediators could provide more information to explain the onset of coronary artery disease than an isolated pathological condition. To investigate this association, they designed the Asymptotic Dental Score, which required a panoramic x-ray to identify signs of odontogenic infection, such as radiolucent periapical areas, long-standing caries, pericoronitis, retained root remnants, vertical bone loss, calculus deposits and restoration with overhangs. They then performed clinical examination to gather information on caries (using the same system as in the Total Dental Index), gingivitis (presence or absence of erythema, bleeding or inflammation), advanced caries or periodontal abscesses as the expression of periapical pathology (0, 1 and ?2), pericoronitis (presence or absence), retained root remnants (0, 1 and ?2) and periodontitis (Community Periodontal Index score of 3 in at least 2 sextants). The final mathematical model, determined by logistic regression analysis, included only 5 variables (pericoronitis, retained root remnants, edentulism, caries and gingivitis), which were given weighted scores (15).

The relationship between infectious pathology of the oral cavity and certain systemic diseases has led to renewed interest in evaluation of the oral health status in the context of routine health controls. As a result, tools that enable us to evaluate the oral health status have gained a certain relevance in recent years, particularly the BOHSE (16). That scale uses 10 variables ( Table 2) that are evaluated by observation and palpation; the only examination instruments required, apart from the obligatory gloves and mask, are a light source, tongue depressors and gauze swabs. The BOHSE was designed specifically as a simple method for auxiliary staff to be able to evaluate the oral health of institutionalised geriatric patients with or without cognitive deterioration; it has subsequently been applied to other collectives, including elderly patients living in the community and institutionalised intellectually-disabled patients (27). This index is a screening tool and cannot therefore be used for diagnostic purposes nor can it replace detailed clinical examination and imaging studies. It is used mainly to detect patients who need to be referred for more complete, specialist dental examination.

A number of variants of the BOHSE have been published, among which special mention should be made of the Oral Health Assessment Tool (OHAT) (28). The main innovations are that the categories “lymph nodes” and “pairs of teeth in occlusion” were eliminated, the category “mucosa of the cheek, floor of the mouth and palate” was combined with the category “interdental gums and/or under prostheses” and a new category, “tooth pain”, was introduced. Although other assessment tools for oral evaluation have been designed, including the Index of Activities of Daily Oral Hygiene (IADOH) and the Mucosal Plaque Score (MPS), a systematic review by Chalmers and Pearson (29) found that the BOHSE was the simplest, most reliable, validated scale for use by nursing staff and carers of institutionalised patients with cognitive deterioration.

One of the main novelties of the present, updated version of the Global Oral Health Scale is quantification of the tooth surfaces that satisfy certain conditions, such as the presence of supragingival plaque, gingivitis and pathological pockets. In this case, the ranges established for the number of surfaces affected correspond to <33%, 33%-66% and >66% of all the surfaces examined. The intervals for the number of caries were based on the values of the DMFT index established by the WHO. The diagnosis of severity of the caries determined by clinical examination can have a large subjective component, particularly when the caries affects the enamel and dentine, but this bias probably becomes less relevant as the number of active caries increases and because of the inclusion of multiple variables in the scale. Periodontal pockets ?4 mm are considered pathological, as has been indicated previously by other authors (30). Other limitations that should be mentioned are that this scale cannot be applied to children under 15 years of age and that its reliability in patients with fewer than 24 teeth has not been established.

To date, using visual inspection of the oral cavity as the reference procedure, we have demonstrated that our objective Global Oral Health Scale is useful to establish a correct diagnosis of oral health in terms of infectious potential (unpublished data). We have also found that our scale shows a positive correlation with the detection and quantification of odontopathogenic and periodontopathogenic bacterial flora present in the saliva, confirming the possible infectious potential (unpublished data).

In conclusion, this scale could be particularly useful for the epidemiological studies comparing different populations and for the analysis of the influence of distinct degrees of oral health on the development of certain systemic diseases.

## References

[1] Albandar JM, Brunelle JA, Kingman A (2002). Destructive periodontal disease in adults 30 years of age and older in the United States, 1988-1994. J Periodontol.

[2] Albandar JM (2005). Epidemiology and risk factors of periodontal diseases. Dent Clin North Am.

[3] Bourgeois DM, Llodra JC, Nordblad A, Pitts NB (2008). Report of the EGOHID I Project. Selecting a coherent set of indicators for monitoring and evaluating oral health in Europe: criteria, methods and results from the EGOHID I project. Community Dent Health.

[4] Montero J, Yarte JM, Bravo M, LÃpez-Valverde A (2011). Oral health-related quality of life of a consecutive sample of Spanish dental patients. Med Oral Patol Oral Cir Bucal.

[5] Baehni PC, Takeuchi Y (2003). Anti-plaque agents in the prevention of biofilm-associated oral diseases. Oral Dis.

[6] Paquette DW (2002). The periodontal infection-systemic disease link: a review of the truth or myth. J Int Acad Periodontol.

[7] Weidlich P, CimÃes R, Pannuti CM, Oppermann RV (2008). Association between periodontal disease and systemic disease. Braz Oral Res.

[8] Bahekar AA, Singh S, Saha S, Molnar J, Arora R (2007). The prevalence and incidence of coronary heart disease is significantly increased in periodontitis: a meta-analysis. Am Heart J.

[9] Zhou X, Wang Z, Song Y, Zhang J, Wang C (2011). Periodontal health and quality of life in patients with chronic obstructive pulmonary disease. Respir Med.

[10] Pizzo G, Guiglia R, Lo Russo L, Campisi G (2010). Dentistry and internal medicine: from de focal infection theory to the periodontal medicine concept. Eur J Intern Med.

[11] Champagne CM, Madianos PN, Lieff S, Murtha AP, Beck JD, Offenbacher S (2000). Periodontal medicine emerging concepts in pregnancy outcomes. J Int Acad Periodontol.

[12] Mattila KJ, Nieminen MS, Valtonen VV, Rasi VP, KesÃniemi YA, SyrjÃlÃ SL (1989). Association between dental health and acute myocardial infarction. BMJ.

[13] Mattila KJ (1993). Dental infections as a risk factor for acute myocardial infarction. Eur Hearth J.

[14] Beck J, Garcia R, Heiss G, Vokonas PS, Offenbacher S (1996). Periodontal disease and cardiovascular disease. J Periodontol.

[15] Janket SJ, QvarnstrÃm M, Meurman JH, Baird AE, Nuutinen P, Jones JA (2004). Asymptotic Dental Score and prevalent coronary heart disease. Circulation.

[16] Kayser-Jones J, Bird WF, Paul SM, Long L, Schell ES (1995). An instrument to assess the oral health status of nursing home residents. Gerontologist.

[17] TomÃs I, Alvarez M, Limeres J, Potel C, Medina J, Diz P (2007). Prevalence, duration and aetiology of bacteraemia following dental extractions. Oral Dis.

[18] Greene JC, Vermillion JR (1964). The Simplified Oral Hygiene Index. J Am Dent Assoc.

[19] Ramfjord SP (1967). The Periodontal Disease Index (PDI). J Periodontol.

[20] LoÃ H, Silness J (1963). Periodontal disease in pregnancy. Acta Odontol Scand.

[21] Barbosa M, Carmona IT, Amaral B, Limeres J, Ãlvarez M, Cerqueira C (2010). General anesthesia increases the risk of bacteremia following dental extractions. Oral Surg Oral Med Oral Pathol Oral Radiol Endod.

[22] OÂLeary TJ, Drake RB, Naylor JE (1972). The plaque control record. J Periodontol.

[23] Ainamo J, Bay I (1975). Problems and proposals for recording gingivitis and plaque. Int Dent J.

[24] Relvas M, Diz P, Velazco C, Otero J, Pacheco JJ, Tomás I Evaluation of partial-mouth recording systems of gingival parameters in a Portuguese adult population. J Public Health Dent.

[25] Grau AJ, Buggle F, Ziegler C, Schwarz W, Meuser J, Tasman AJ (1997). Association between acute cerebrovascular ischemia and chronic and recurrent infection. Stroke.

[26] Oikarinen K, Zubaid M, Thalib L, Soikkonen K, Rashed W, Lie T (2009). Infectious dental diseases in patients with coronary artery disease: an orthopantomographic case-control study. J Can Dent Assoc.

[27] Chen CC, Chang CK, Chyun D, McCorkle R (2005). Dynamics of nutritional health in a community sample of american elders: a multidimensional approach using roy adaptation model. ANS Adv Nurs Sci.

[28] Chalmers JM, King PL, Spencer AJ, Wright FA, Carter KD (2005). The Oral Health Assessment Tool â Validity and reliability. Aust Dent J.

[29] Chalmers JM, Pearson A (2005). A systematic review of oral health assessment by nurses and carers for residents with dementia in residential care facilities. Spec Care Dentist.

[30] Querna JC, Rossmann JA, Kerns DG (1994). Prevalence of periodontal disease in an active duty military population as indicated by an experimental periodontal index. Mil Med.

